# Various Viewpoints to Investigate the Origins of Life Are Needed

**DOI:** 10.3390/life14101324

**Published:** 2024-10-18

**Authors:** Tony Z. Jia, Kuhan Chandru

**Affiliations:** 1 Earth-Life Science Institute, Institute of Future Science, Institute of Science Tokyo, 2-12-1-IE-1 Ookayama, Meguro-ku, Tokyo 152-8550, Japan; 2 Blue Marble Space Institute of Science, 600 1st Ave., Floor 1, Seattle, WA 98104, USA; 3 Space Science Center (ANGKASA), Institute of Climate Change, National University of Malaysia, Bangi 43650, Selangor, Malaysia; 4 Polymer Research Center (PORCE), Faculty of Science and Technology, National University of Malaysia, Bangi 43600, Selangor, Malaysia; 5 Institute of Physical Chemistry, CENIDE, University of Duisburg-Essen, 45141 Essen, Germany

How life first arose on Earth is a mystery that humankind has sought to understand for millennia, and includes scientific, philosophical, societal, and religious aspects, amongst others. Even more mysterious is whether life exists elsewhere, including whether the conditions for life to arise exist beyond Earth. While it is possible to discuss these topics from any viewpoint, as researchers, we seek to answer these questions from a scientific perspective, which requires rigorous investigation in a variety of fields, from astronomy to planetary science, geology, physics, chemistry, biology, and others. To that end, a Special Issue featuring contributions from all aspects of origins of life (OoL) research was set up to highlight the diversity of the field and the breadth of the knowledge required to even attempt to understand OoL on Earth and elsewhere, and also to illuminate unanswered questions that will be tackled by the next generation of OoL researchers. This Special Issue was guest-edited by Paul Higgs, Addy Pross, Kuhan Chandru, and Tony Z. Jia (the latter two being the authors of this Editorial) Here, we briefly highlight the 19 contributions to this Special Issue as arranged in a broad range of research subtopics. Many papers could belong to more than one subtopic, which shows the interdisciplinarity of the field, and the breadth of topics that the contributions covered suggests the importance of incorporating various viewpoints when considering questions related to OoL.

Potiszil et al. review what we currently know about organics in Ryugu, an asteroid which the Hayabusa2 mission mined to bring samples back to Earth for analysis. They synthesize known information and gives context to theories as to how Ryugu formed, while also focusing on the possibility of future analyses of Ryugu samples [1]. Ishikawa et al. experimentally study how gamma rays could lead to the formation of amino acids within carbonaceous chondrites. They report that increasing doses of gamma radiation of simple organic starting materials lead to the enhanced formation of various amino acids, most notably alanine and glycine, and that gamma radiation enhances the formation of glycoaldehyde from formaldehyde starting materials, a key step in amino acid formation [2].

Lei and Burton review the 3 31 nt minihelix tRNA evolution theorem and propose its acceptance as a proven theory in biology. They discuss statistical, functional, and structural reasons that support this theorem, placing tRNA at the center of OoL [3]. Schoenmakers et al. review the application of evolution theory to the origins of life, specifically focusing on how evolution can be applied to prebiotic chemistry, and, in particular, metabolism. They combine discussion and analysis not only from a scientific viewpoint, but also from philosophy, simultaneously highlighting unanswered questions in the field [4]. Brunk and Marshall review how reflexively autocatalytic food-generated networks led to the origin of eukaryotic cells. In particular, they suggest that because bacteria and archaea are so different, cellularization likely evolved twice, eventually leading to the emergence of eukaryotes via the fusion of bacteria and archaea [5]. Yarus presents a computational study which investigates the origin of the standard genetic code. According to this study, code division results in rapid code evolution, while the combination of fusion and division further imbues accuracy into rapid code evolution, suggesting that a combination of fusion and division, both standard cellular processes, likely led to the origin of the standard genetic code [6]. Mayer et al. study order and complexity in evolving micellar systems through simulations based on the graded autocatalysis replication domain (GARD) model. They report that lipid-based micellar and vesicular systems increase in order and complexity over time, which signifies their ability to undergo Darwinian evolution and potentially makes them key structures at the origins of life [7].

Brown et al. review the importance of non-canonical amino acids at the origins of life, providing structural, chemical, and functional evidence that suggests the significance of xeno amino acids early on in biology. They then allude to a number of unanswered questions that can be taken up by the next generation of researchers [8]. Agmon reviews the contribution of three biopolymer types, DNA, RNA, and proteins, to the origins of life. Specifically, the author suggests that RNA and proteins likely had to co-evolve in order to jumpstart the origins of life [9]. Weller reviews the plausibility of how the first homochiral molecules on Earth initially arose. The author suggests that rather than positing the existence of a homochiral monomer pool, there is a statistical possibility that homochiral polymers could have formed from a non-enantiopure chemical milieu [10]. Chandru et al. review how contingency could have played a role at the origins of life. In particular, they discuss how non-biomolecules, i.e., those that do not participate significantly in modern biology, could have led to the origins of life both on and off Earth [11]. Roche et al. present research showing the plausibility that proto-nucleotides can be synthesized in a single one-pot reaction containing proto-nuclebases (barbituric acid and melamine) and ribose-5-phosphate. These proto-nucleotides then self-assemble into structures showing Watson–Crick basepairing ability, suggesting one mechanism by which proto-RNA could have emerged on early Earth [12]. Cowan presents a computational study investigating how the weak nuclear force can lead to autocatalytic amino acid synthesis. According to this study, a metal-promoted Strecker mechanism could lead to the emergence of a homochiral pool of amino acid monomers [13]. Lopez et al. experimentally investigate conditions conducive to the progression of key oligonucleotide and peptide-based primitive reactions in conditions favorable to lipid vesicle formation. Given that specific conditions simultaneously optimal to all of these processes could not be discovered in this study, the authors propose that nucleic acids and peptides evolved independently at the origins of life [14]. Verma et al. employ microfluidic devices to generate phase transitions driven by the self-assembly of a small molecule dye. Using the microfluidic devices, they are able to recapitulate the phase diagram of a liquid crystalline phase transition with the automation of dehydration–rehydration processes. They propose incorporating more microfluidics technologies into origins of life research due to their ability to rapidly manipulate small volumes of liquids, leading to higher experimental throughput with less waste [15].

Fox et al. present computational research showing how salt-induced peptide formation could have led to the origin of homochirality. Specifically, density functional theory calculations found no evidence that the geometry of the CuCl active complex led to the stereoselectivity of peptide formation, which opens up other possible mechanisms that the field is challenged to investigate [16]. Daga et al. use a numerical model to simulate how changes in environmental conditions affect evolving protocells. In particular, they find that in stable environments, evolving protocells outcompete non-evolving protocells. However, when environmental changes occur on a similar timescale to the lifetime of a single generation, evolving protocells are outcompeted, likely due to energetic cost from their optimization for different conditions [17]. Ravanbodshirazi et al. investigate how various experimental conditions can affect spark discharge reaction product distribution. They find that while temperature has an impact, product distribution is most greatly affected by how the spark itself is generated, including the type of equipment used [18]. Finally, Christensen et al. use a thermochemical model to estimate the formation of atmospheric NO and HCN and an updated photochemical model to estimate fixed nitrogen species production on Hadean Earth. The updated model includes a new radical-based pathway to generate HCN, which results in the estimation of greater levels of HCN than previously found [19].
